# A novel caged Cookson‐type reagent toward a practical vitamin D derivatization method for mass spectrometric analyses

**DOI:** 10.1002/rcm.8648

**Published:** 2020-02-19

**Authors:** Masahiko Seki, Makoto Sato, Masaki Takiwaki, Koji Takahashi, Yoshikuni Kikutani, Mamoru Satoh, Fumio Nomura, Yutaka Kuroda, Seketsu Fukuzawa

**Affiliations:** ^1^ Medical Association Group Tokuyama Corporation Ibaraki Japan; ^2^ Tsukuba Research Lab Tokuyama Corporation Ibaraki Japan; ^3^ Open Innovation Promotion Department, Management Strategy Planning Division JEOL Ltd. Tokyo Japan; ^4^ Division of Clinical Mass Spectrometry Chiba University Hospital Chiba Japan; ^5^ Department of Biotechnology and Life Science, Faculty of Technology Tokyo University of Agriculture and Technology Tokyo Japan

**Keywords:** Cookson, vitamin D, clinical laboratory, mass spectrometry, derivatization

## Abstract

**Rationale:**

25‐Hydroxylated vitamin D is the best marker for vitamin D (VD). Due to its low ionization efficiency, a Cookson‐type reagent, 1,2,4‐triazoline‐3,5‐dione (TAD), is used to improve the detection/quantification of VD metabolites by liquid chromatography/tandem mass spectrometry (LC/MS/MS). However, the high reactivity of TAD makes its solution stability low and inconvenient for practical use. We here describe the development of a novel caged Cookson‐type reagent, and we assess its performances in the quantitative and differential detection of four VD metabolites in serum using LC/MS/MS.

**Methods:**

Caged 4‐(4′‐dimethylaminophenyl)‐1,2,4‐triazoline‐3,5‐dione (DAPTAD) analogues were prepared from 4‐(4′‐dimethylaminophenyl)‐1,2,4‐triazolidine‐3,5‐dione. Their stability and reactivity were examined. The optimized caged DAPTAD (14‐(4‐(dimethylamino)phenyl)‐9‐phenyl‐9,10‐dihydro‐9,10‐[1,2]epitriazoloanthracene‐13,15‐dione, DAP‐PA) was used for LC/MS/MS analyses of VD metabolites.

**Results:**

The solution stability of DAP‐PA in ethyl acetate dramatically improved compared with that of the non‐caged one. We measured the thermal retro‐Diels‐Alder reaction enabling the release of DAPTAD and found that the derivatization reaction was temperature‐dependent. We also determined the detection limit and the lower limit of quantifications for four VD metabolites with DAPTAD derivatization.

**Conclusions:**

DAP‐PA was stable enough for mid‐ to long‐term storage in solution. This advantage shall contribute to the detection and quantification of VD in clinical laboratories, and as such to the broader use of clinical mass spectrometry.

## 1 INTRODUCTION

Vitamin D (VD) metabolites play an important role in homeostatic maintenance, such as bone metabolism.^1^ Because VD deficiency is reportedly linked to a wide range of human diseases, the requirement for the evaluation of VD status is increasing in clinical medicine.^1^ 25‐Hydroxylated VD (25(OH)D) is the best marker for VD status.^2^ Although 25‐hydroxyvitamin D_3_ (25(OH)D_3_) normally accounts for most of serum 25(OH)D, differential determination of 25‐hydroxyvitamin D_2_ (25(OH)D_2_) is desirable when VD_2_‐containing supplements are used by the subject (patient). Because significant levels of the C‐3 epimer of 25(OH)D_3_, 3‐*epi*‐25(OH)D_3_, are present in the serum in both infants and adults,^3^ its presence may result in the overestimation of 25(OH)D if it is not properly resolved by chromatography. Furthermore, the accurate quantification of 24*R*,25‐dihydroxyvitamin D_3_ (24,25(OH)_2_D_3_) is essential for the differential diagnosis of infantile hypercalcemia of unknown etiology.^4^ Thus, there is an increasing demand for the quantitative and routine mass spectrometric measurement of differential VD metabolites.^5^ Because VD metabolites exhibit a low ionization efficiency under the conditions used in VD analysis by liquid chromatography/tandem mass spectrometry (LC/MS/MS), attempts to improve ionization efficiency by derivatization are being reported.^6^

**Figure 1:**
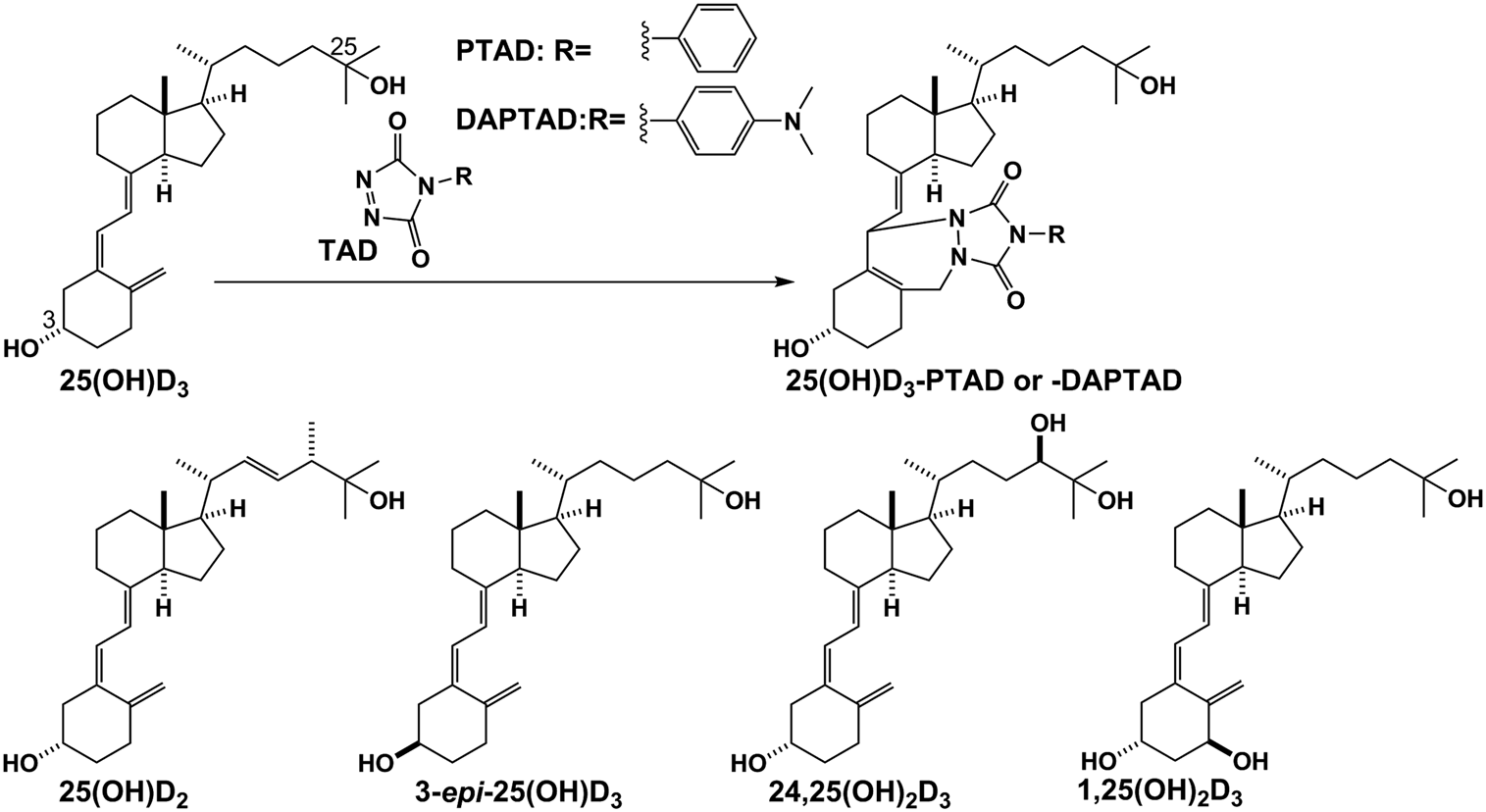
Derivatization of vitamin D (VD) metabolites using Cookson‐type reagents

VD metabolites have a particular structural feature: a conjugated *s‐cis* diene. Consequently, VD selective derivatization reagents that take advantage of the reactive dienophile, 1,2,4‐triazoline‐3,5‐dione (TAD), which is known as a Cookson‐type reagent, have been developed for enhancing their detection limits in mass spectrometric analyses.^7^, ^8^ 4‐Phenyl‐1,2,4‐triazoline‐3,5‐dione (PTAD)^9^ is a well‐known VD metabolite selective derivatization reagent for LC/MS/MS, and its improved version, 4‐(4′‐dimethylaminophenyl)‐1,2,4‐triazoline‐3,5‐dione (DAPTAD),^10^, ^11^ developed by Higashi et al., enables a quantitative and differential detection of VD metabolites (Figure 1). We previously described the quantitative measurement of four VD metabolites (25(OH)D_3_, 25(OH)D_2_, 3‐*epi*‐25(OH)D_3_, and 24,25(OH)_2_D_3_) in serum by LC/MS/MS using DAPTAD derivatization.^12^ DAPTAD, however, is not necessarily appropriate for routine use in the clinical laboratory because of the following reasons: it is difficult to isolate DAPTAD from the reaction mixture; DAPTAD solution without purification needs to be stored at −18°C and is recommended for use within 2 months.^10^ In addition, on‐site preparation of the chemicals is inherently associated with a risk of contamination, which may affect the yield of the derivatization reaction. Furthermore, water condensation can also disturb or hamper the derivatization reaction, and ambient or near‐ambient temperature (refrigerator level) storage of the reagent solution is thus desirable, which, on the other hand, will affect the reagent's stability. Overcoming these limitations would enable not only an easier operational handling, but may also open the way to the automated and routine detection/quantification of VD metabolites. Effective TAD reagents, including DAPTAD, for VD metabolites have been reported;^13^, ^14^ however, their solution stability were often low or in many cases not investigated. Although 2‐nitrosopyridine^15^ might be a better derivatization reagent for VD metabolites than TAD reagents, popularity and accumulated knowledge of 2‐nitrosopyridine as a derivatization reagent are still limited compared with those of well‐known and widely used TAD reagents. In addition, long‐term solution stability of 2‐nitrosopyridine has not yet been confirmed. These factors motivated us to use TAD in our experiment. The stability of TAD reagents is dependent on the TAD group and not on the conjugated aromatic functional group. Therefore, we focused on DAPTAD as a representative TAD molecule.

We describe the development of a novel caged Cookson‐type reagent, 14‐(4‐(dimethylamino)phenyl)‐9‐phenyl‐9,10‐dihydro‐9,10‐[1,2]epitriazoloanthracene‐13,15‐dione (DAP‐PA), which can produce DAPTAD by thermal retro‐Diels‐Alder (RDA) reaction and present preliminary analytical performances of DAP‐PA when applied to the quantitative and differential measurement of the four VD metabolites in serum by LC/MS/MS.

## 2 EXPERIMENTAL

### 2.1 Characterization of synthetic compounds

^1^H‐NMR (nuclear magnetic resonance) spectra were recorded on a JEOL JNM‐ECA400II and a JEOL JNM‐ECA500 spectrometer (Akishima, Tokyo, Japan) operating at 400 MHz (^1^H) and 100 MHz (^13^C), respectively. Chemical shifts are reported in δ units and referenced to the solvent, that is, 7.24 (^1^H) and 77.0 (^13^C) for CDCl_3_. Multiplicities are indicated as br (broadened), s (singlet), d (doublet), t (triplet), or m (multiplet). Coupling constants (*J*) are reported in Hertz (Hz). High‐resolution mass spectra (HRMS) were recorded on a JEOL T100LP mass spectrometer (Akishima, Tokyo, Japan) under fast atom bombardment (FAB) conditions using *m*‐nitrobenzyl alcohol as a matrix. IR spectra were recorded on a Spectrum One spectrometer (Perkin Elmer, Waltham, MA, USA).

### 2.2 Preparation of caged DAPTAD

**Figure 2:**
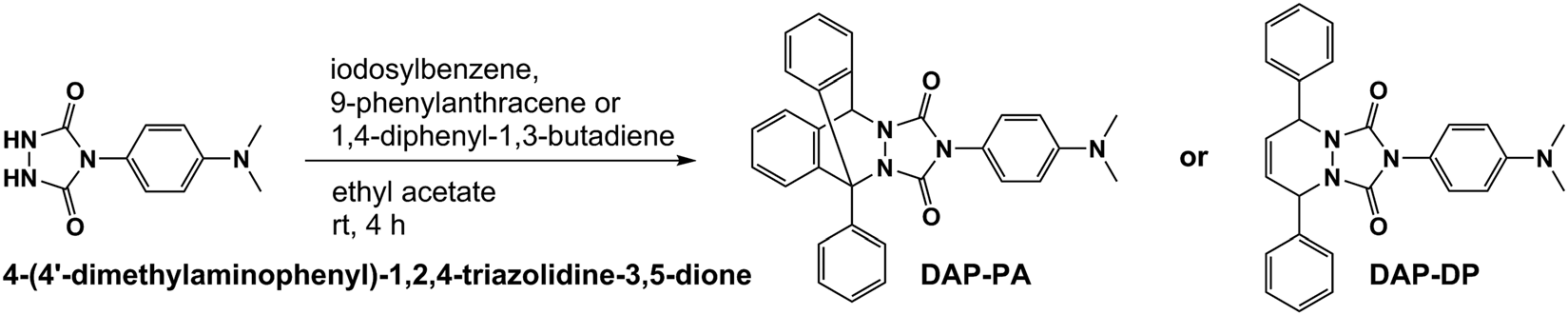
Preparation of caged 4‐(4′‐dimethylaminophenyl)‐1,2,4‐triazoline‐3,5‐dione (DAPTAD)

4‐(4′‐Dimethylaminophenyl)‐1,2,4‐triazolidine‐3,5‐dione^10^ (0.5 g, 2.27 mmol), iodosylbenzene (0.5 g, 2.27 mmol), anhydrous magnesium sulfate (2 g), and 9‐phenylanthracene or 1,4‐diphenyl‐1,3‐butadiene (1 eq) were added to a nitrogen‐purged ethyl acetate solution (Figure 2). The resultant reaction mixture was stirred at room temperature in the dark until the solution turned dark red. After 4 h, the mixture was transferred into a separation funnel, washed with 5% sodium hydrogen carbonate (80 mL), and again washed with water (80 mL × 2) and brine. The organic layer was dried over anhydrous magnesium sulfate, filtrated, and concentrated under reduced pressure. Purification by silica gel chromatography (hexane/ethyl acetate [7:3, v/v]) produced the caged DAPTAD as white crystals. The yields were 52% for the 9‐phenylanthracene adduct (DAP‐PA) and 61% for the 1,4‐diphenyl‐1,3‐butadiene adduct (2‐(4‐(dimethylamino)phenyl)‐5,8‐diphenyl‐5,8‐dihydro‐1*H*‐[1,2,4]triazolo[1,2‐*a*]pyridazine‐1,3(2*H*)‐dione, DAP‐DP).

### 2.3 Physical properties of DAP‐PA

^1^H‐NMR (CDCl_3_) δ 7.85 (d, *J* = 7.6 Hz, 2H), 7.59–7.49 (m, 5H), 7.31 (td, *J* = 7.6, 1.2 Hz, 2H), 7.22 (td, *J* = 7.6, 1.2 Hz, 2H), 7.03 (d, *J* = 7.6 Hz, 2H), 6.78 (ddd, *J* = 9.0, 3.2, 2.4 Hz, 2H), 6.56 (ddd, *J* = 9.0, 3.2, 2.4 Hz, 2H), 6.40 (s, 4H), 2.87 (s, 6H); ^13^C‐NMR (CDCl_3_) δ 158.2, 157.3, 150.3, 139.3, 132.5, 129.8, 128.8, 128.1, 128.0, 127.8, 126.6, 124.5, 123.9, 119.4, 112.2, 72.6, 62.6, 40.5; IR (KBr, cm^−1^) 3062, 1774, 1714, 1613, 1526, 1460, 1448, 1417, 1361, 1248, 1230, 1181, 1158, 1039, 1004, 946, 846, 805, 784, 764, 753, 702, 679, 634, 541; HRMS (FAB) calculated for C_30_H_25_N_4_O_2_ [(M + H)^+^] 473.19775, found 473.19683.

### 2.4 Physical properties of DAP‐DP

^1^H‐NMR (CDCl_3_) δ 7.52 (brd, *J* = 8.4 Hz, 4H), 7.42–7.32 (m, 6H), 7.18 (ddd, *J* = 9.2, 3.2, 2.4 Hz, 2H), 6.63 (ddd, *J* = 9.2, 3.2, 2.4 Hz, 2H), 5.99 (d, *J* = 1.2 Hz, 2H), 5.53 (d, *J* = 1.2 Hz, 2H), 2.89 (s, 6H); ^13^C‐NMR (CDCl_3_) δ 152.6, 137.4, 128.8, 128.6, 127.9, 126.4, 125.4, 112.3, 58.3, 40.5; IR (KBr, cm^−1^) 3031, 2941, 2802, 1771, 1701, 1614, 1523, 1493, 1447, 1420, 1352, 1289, 1193, 1158, 1143, 1063, 946, 878, 846, 819, 753, 698, 610, 588, 517; HRMS (FAB) calculated for C_26_H_25_N_4_O_2_ [(M + H)^+^] 425.19775, found 425.18956.

### 2.5 Analytical high‐performance liquid chromatography (HPLC)

The chromatographic analysis of the RDA reaction was performed using an Alliance HPLC system equipped with a model 2489 UV–Vis detector (Waters, Milford, MA, USA). Chromatography was performed using an Inertsil ODS‐3 column (4.6 mm I.D. × 250 mm (5 μm; GL Sciences, Tokyo, Japan) with a binary gradient system with water (A) and acetonitrile (B) and a flow rate of 1.0 mL/min. The detection wavelength was 210 nm, and the elution program was as follows: 0 → 10 min, 50% B; 10 → 20 min, 50 → 5% B; 20 → 50 min, 5% B; 50 → 55 min, 50% B; 55 → 60 min, 50% B.

### 2.6 Kinetic analysis of the RDA reaction

**Figure 3:**
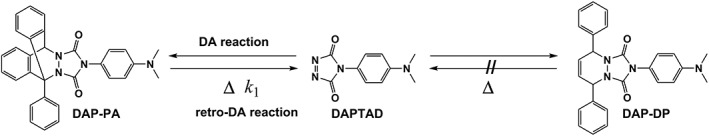
Thermal retro‐Diels‐Alder (RDA) reaction of the caged DAPTAD

**Figure 4:**
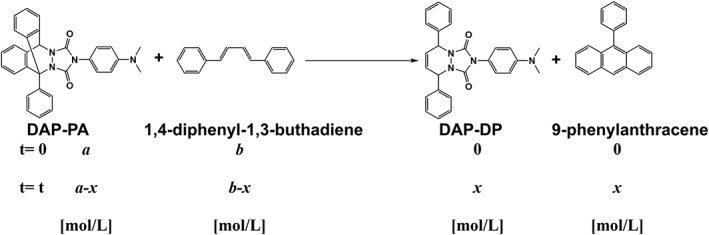
Kinetic analysis of the RDA reaction

The rate constant *k*_1_ was determined using the following equation describing the irreversible diene tag, 1,4‐diphenyl‐1,3‐buthadiene (Figures 3 and 4).

$$dx/dt=k_{1}\left(a–x\right).$$

$$\ln\;a/\left(a–x\right)=k_{1}\;t.$$

### 2.7 Materials for LC/MS/MS

The JeoQuant kit for LC/MS/MS analysis of vitamin D metabolites including calibrators (25(OH)D_3_, 25(OH)D_2_, 3‐*epi*‐25(OH)D_3_, and 24,25(OH)_2_D_3_); internal standards (25(OH)D_3_‐[23,24,25,26,27‐^13^C_5_], 25(OH)D_2_‐[25,26,27‐^13^C_3_], 3‐*epi*‐25(OH)D_3_‐[23,24,25,26,27‐^13^C_5_], and 24,25(OH)_2_D_3_‐[26,26,26,27,27,27‐^2^H_6_]); quality controls; and the derivatization reagent (DAP‐PA ethyl acetate solution [0.5 mg/mL]) were obtained from JEOL (Akishima, Tokyo, Japan; https://www.jeol.co.jp/en/support/support_system/contact_jeoquantkit.html). The calibrator concentrations for 25(OH)D_3_, 25(OH)D_2_, 3‐*epi*‐25(OH)D_3_, and 24,25(OH)_2_D_3_ were as follows: 0.873, 0.0920, 0.0940, and 0.0960 ng/mL for calibrator level 1; 8.80, 0.924, 0.938, and 0.962 ng/mL for calibrator level 2; 44.0, 4.62, 4.69, and 4.81 ng/mL for calibrator level 3; and 88.0, 9.23, 9.37, and 9.61 ng/mL for calibrator level 4. These concentrations were determined by quantitative NMR measurements.^16^ Human Serum Standard Reference Material (SRM972a) was obtained from the National Institute of Standards and Technology (NIST, Gaithersburg, MD, USA). LC/MS‐grade acetonitrile and formic acid were purchased from Wako Pure Chemical Industries (Osaka, Japan). HPLC‐grade distilled water was obtained from Nacalai Tesque (Kyoto, Japan).

### 2.8 DAPTAD derivatization

DAP‐PA solution (100 μL) was added to the dried samples, and the resultant mixture was heated at 80°C for 15 min. Ethanol (20 μL) was added to the mixture to terminate the reaction, and the solvent was removed by centrifuged evaporation. The residue was dissolved in 50 μL of 30% aqueous acetonitrile for LC/MS/MS analyses.

### 2.9 Extraction of VD metabolites from SRM972a level 2 serum

SRM972a level 2 serum (50 μL) and internal standards (250 μL) were mixed and vortexed for 1 min. Each sample was loaded into a supported liquid extraction column (ISOLUTE® SLE+ 400 μL sample volume, Biotage, Uppsala, Sweden) and eluted three times with 600 μL ethyl acetate/hexane (1:1, v/v) using a PRESSURE+48 positive pressure manifold (Biotage). The combined eluents were evaporated to dryness using a centrifuged evaporator. DAPTAD derivatization of the obtained samples was carried out as described above.

### 2.10 LC/MS/MS analyses

LC/MS/MS analyses were performed using an QTRAP4500® triple quadruple mass spectrometer (AB SCIEX, Framingham, MA, USA) equipped with a model 1290 Infinity liquid chromatography system (Agilent Technologies, Santa Clara, CA, USA). Chromatography was performed using a binary gradient system with 0.1% formic acid (A) and 0.1% formic acid in acetonitrile (B) or methanol (C). Samples were dissolved in 50 μL of 30% aqueous acetonitrile and loaded into a CAPCELL CORE C_18_ (2.7 μm) 2.1 mm I.D. × 100 mm column (OSAKA SODA, Osaka, Japan) equilibrated with 0.1% formic acid 30% acetonitrile with a flow rate of 0.3 mL/min. The injection volume was 20 μL. The elution program was as follows: 0.01 → 0.5 min, 30 → 58% B; 0.5 → 7.0 min, 58% B; 7.0 → 7.01 min, 58 → 90% B; 7.01 → 8.0 min, 90% B; 8.0 → 8.01 min, 90 → 30% B; 8.01 → 9.0 min, 30% B. For analyses of non‐derivatized VD metabolites, an InertSustain PFP HP (3 μm) 2.1 mm I.D. × 150 mm column (GL Sciences, Tokyo, Japan) was used. The flow rate was 0.4 mL/min, and the elution program was as follows: 0.01 → 0.5 min, 50 → 67% C; 0.5 → 9.0 min, 67% C; 9.0 → 9.01 min, 67 → 90% C; 9.01 → 11.0 min, 90% C; 11.0 → 11.01 min, 90 → 50% C; 11.01 → 13.0 min, 50% C. Ionization was done with the positive ion mode, and selected reaction monitoring (SRM) was used for quantification. The transitions (*m/z*) and collision energies (CE) for the VD metabolites are summarized in Table 1. Operation and quantification analyses were done using Analyst® (version 1.7) and SCIEX OS software (AB SCIEX), respectively. The limit of detection (LOD) was determined from duplicate measurements of the authentic VD metabolite solution diluted with water as follows: 1:1 (not diluted), 1:8, 1:16, 1:32, 1:64, 1:128, 1:200, 1:256, 1:400, and 1:800. The LODs were defined as the concentration of VD metabolites per injection giving a signal‐to‐noise ratio (S/N) of 10, which was manually calculated as the peak height divided by the noise level around the peak. The lower limit of quantification (LLOQ) was determined from three replicate measurements of SRM972a level 2 serum diluted with water as follows: 1:1 (not diluted), 1:2, 1:4, 1:8, 1:10, 1:16, 1:20, 1:32, 1:40, 1:64, 1:80, 1:160, 1:320, and 1:640. The LLOQs were set to the minimum level at which dilution linearity was confirmed and the coefficient of the variance was at most 15% and given by the concentration of VD metabolites per injection giving the S/N of 15.

**Table 1:** Tandem mass spectrometry parameters of vitamin D (VD) metabolites with and without 4‐(4′‐dimethylaminophenyl)‐1,2,4‐triazoline‐3,5‐dione (DAPTAD) derivatization

	With derivatization		Without derivatization	
	*m/z*	CE	*m/z*	CE
25(OH)D_3_ and 3‐*epi*‐25(OH)D_3_	619.3 > 341.1	35	401.2 > 91.0	89
25(OH)D_3_–^13^C_5_ and 3‐*epi*‐25(OH)D_3_–^13^C_5_	624.5 > 341.1	35		
25(OH)D_2_	631.3 > 341.1	33	417.1 > 105.1	65
25(OH)D_2_‐^13^C_3_	634.3 > 341.2	33		
24,25(OH)_2_D_3_	635.3 > 341.1	37	417.1 > 105.1	65
24,25(OH)_2_D_3_‐*d*_6_	641.5 > 341.1	37		

CE, collision energy.

## 3 RESULTS AND DISCUSSION

### 3.1 Solution stability of DAPTAD and DAP‐PA

**Figure 5:**
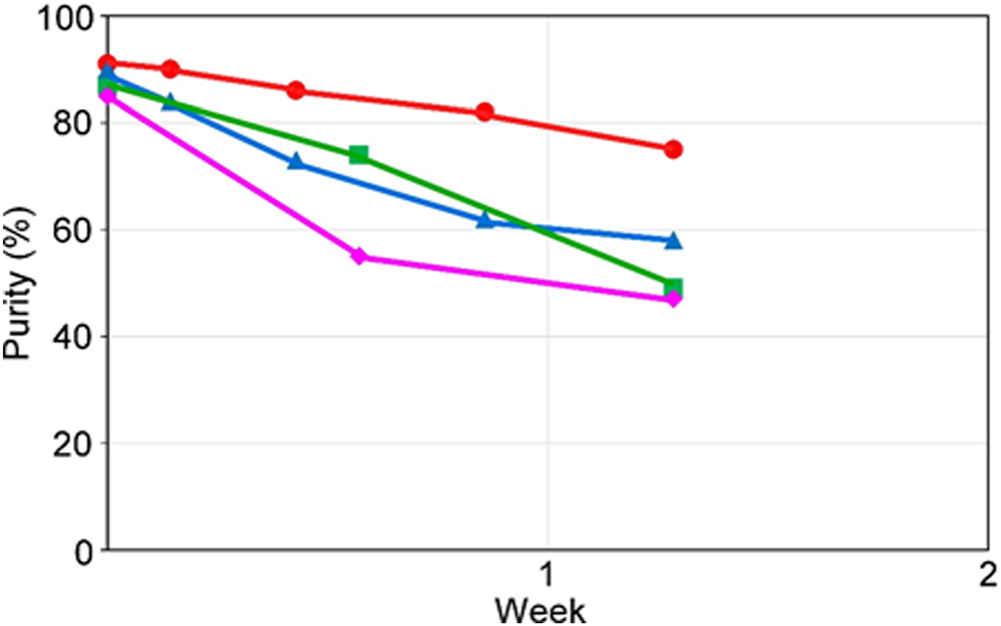
Solution stability of DAPTAD in ethyl acetate‐*d*_8_ with (●: 4°C; ▲: 25°C) and without (■: 4°C; ◆: 25°C) molecular sieves 4A monitored using NMR spectroscopy [Color figure can be viewed at http://wileyonlinelibrary.com]

**Figure 6:**
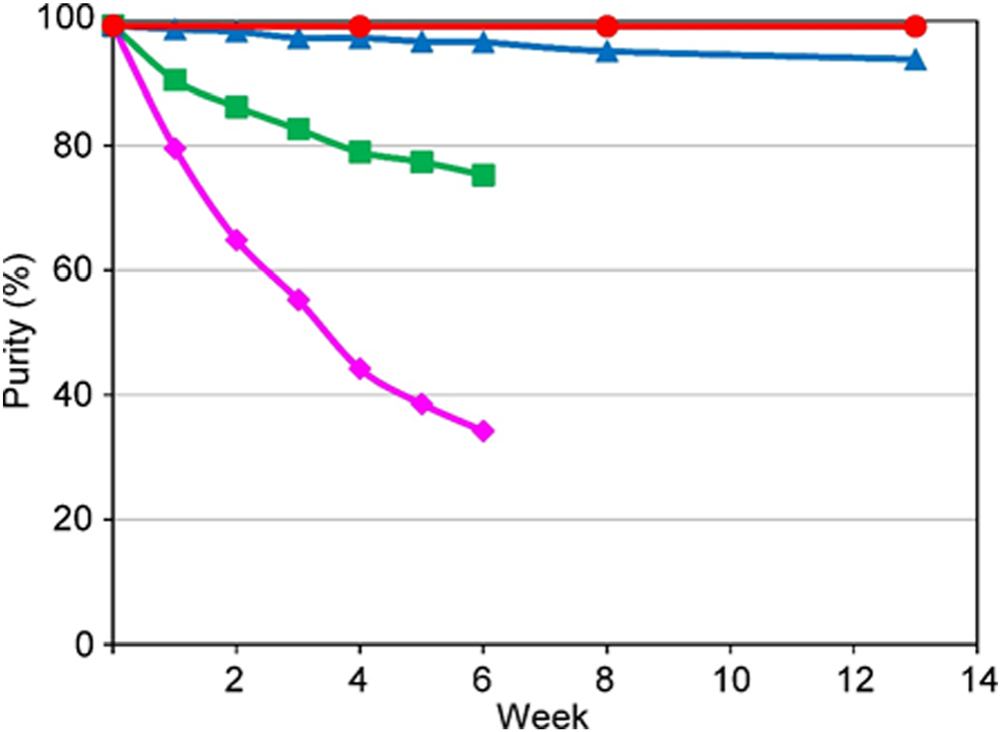
Solution stability of DAP‐PA monitored by HPLC. –22.5°C (●), 6.8°C (▲), 25°C (■), 40°C (◆) [Color figure can be viewed at http://wileyonlinelibrary.com]

Although TAD is one of the most reactive dienophiles and provides an excellent derivatization tag, its solution instability has been reported.^17^, ^18^, ^19^, ^20^ In nucleophilic solvent systems (alcohols or water), it undergoes a nucleophilic attack of its oxygen functional groups or a loss of nitrogen‐yielding dimeric compounds. We first examined the solution stability of DAPTAD, which was synthesized according to the published protocol,^10^ using NMR. The amount of residual DAPTAD was estimated by the integration of aromatic proton peaks (Figures 5 and S1, supporting information). As expected, a time‐dependent decrease in the proton signals was observed in ethyl acetate, which is a common solvent for DAPTAD derivatization.^10^ The addition of molecular sieves 4A at 4°C increased the solution stability of DAPTAD, which strongly suggested that a nucleophilic attack by residual moisture occurred in the non‐caged TAD. On the other hand, the solution stability of DAP‐PA in ethyl acetate dramatically improved (Figure 6) in comparison with that of the non‐caged TAD, which indicated that a DA‐type protection (Figure 3) of the dienophile, TAD, was effective.

### 3.2 RDA reaction of caged DAPTAD

The RDA reaction is a reversible reaction, which can be controlled by thermal regulation (Figure 3).^21^, ^22^ This dynamic, reversible covalent bond formation and cleavage makes it possible to consider thermal protection and deprotection. The thermal RDA reaction enabling the release of DAPTAD was examined using analytical HPLC. DAPTAD itself was difficult to detect using reversed‐phase HPLC due to its instability in aqueous solvents, and we thus monitored the presence of 1,4‐diphenyl‐1,3‐butadiene and 9‐phenylanthracene at 70°C in ethyl acetate, which showed that the reaction with 1,4‐diphenyl‐1,3‐butadiene was irreversible (Figure 3). On the other hand, the release of 9‐phenylanthracene was observed using the irreversible diene tag, 1,4‐diphenyl‐1,3‐buthadiene (Figures 4 and S8, supporting information). The release rate of 9‐phenylanthracene from DAP‐PA in ethyl acetate was temperature‐dependent (Table 2).^21^, ^22^ The reaction temperature (Table S9, supporting information), time (Table S10, supporting information), solvent (Table S2, supporting information), and concentration (Figure S11, supporting information) were also investigated using LC/MS/MS. The reaction was saturated in about 15 min. Although the reaction with aromatic solvents (toluene, anisole, and *o*‐dichlorobenzene) gave better rate constants than that with ethyl acetate (Table S2, supporting information), the peak area in the SRM chromatogram of derivatized VD metabolites produced in ethyl acetate was larger than that produced in toluene (Figure S11, supporting information). Considering their boiling points (indicating the easiness to remove), we concluded that ethyl acetate at 80°C was the best reaction condition.

**Table 2:** Release rate of 9‐phenylanthracene from DAP‐PA in ethyl acetate

Reaction temperature (°C)	Rate constant *k*_1_ (h^−1^)
50	0.07
60	0.18
70	1.01
77^a^	2.50

a Boiling point of ethyl acetate.

### 3.3 LC/MS/MS analyses of VDs without serum

Four VD metabolites with and without DAPTAD derivatization in the absence of serum were detected using the previously reported procedure.^12^ Their retention time (*t*_R_), LOD values, and sensitivity increase are summarized in Table 3.

**Table 3:** LODs for VD metabolites with and without DAPTAD derivatization in the absence of serum

	With derivatization		Without derivatization		
	*t*_R_ a (min)	LOD (ng/mL)	*t*_R_ a (min)	LOD (ng/mL)	Sensitivity increase
25(OH)D_3_	5.59	0.055	6.97	1.4	25
25(OH)D_2_	6.70	0.036	7.60	4.6	128
3‐*epi*‐25(OH)D_3_	5.27	0.037	7.49	0.59	16
24,25(OH) _2_D_3_	2.97	0.037	4.26	0.30	8

LOD, limit of detection.

a Derivatized and non‐derivatized VD metabolites were analyzed using CAPCELL CORE C_18_ and InertSustain PFP HP columns, respectively.

### 3.4 LC/MS/MS analyses of VDs in SRM972a level 2 serum

**Figure 7:**
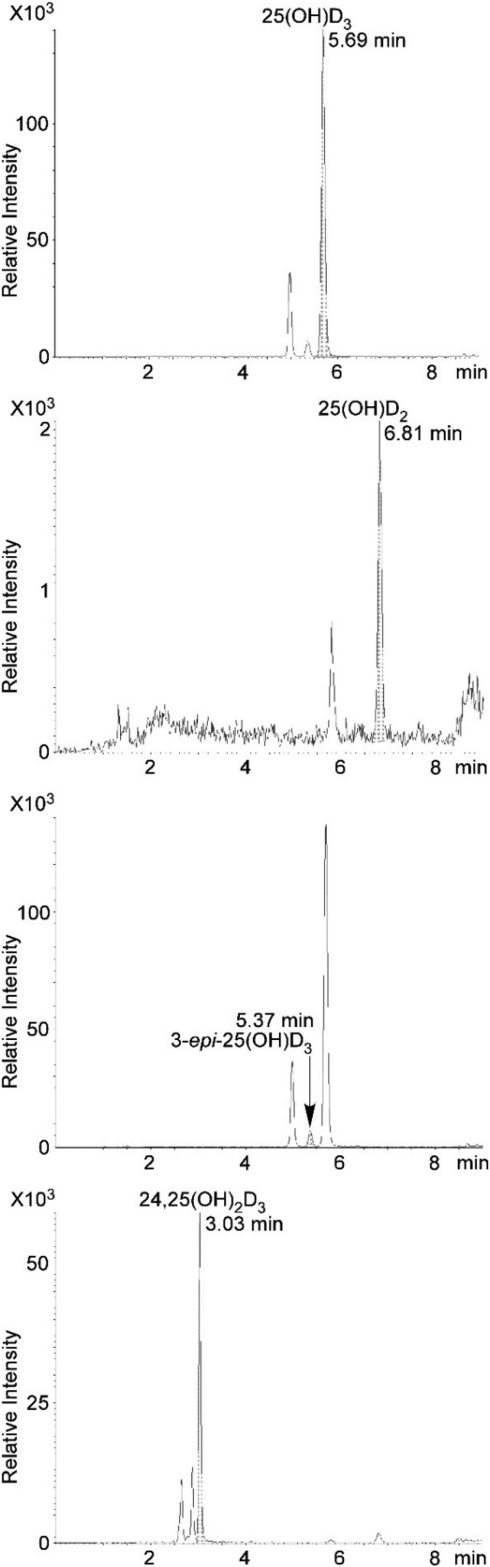
Selected reaction monitoring chromatograms of DAPTAD‐derivatized VDs in SRM972a level 2 serum

LLOQs of four VD metabolites in SRM972a level 2 serum with DAPTAD derivatization are summarized in Table 4. Their SRM chromatograms are shown in Figure 7. In our study 1α, 25‐dihydroxyvitamin D_3_ (1,25(OH)_2_D_3_) could not be detected quantitatively due to its extremely low concentration and the difficulty in chromatographic separation of 1,25(OH)_2_D_3_ and other dihydroxylated VD metabolites such as 4β, 25‐dihydroxyvitamin D.^23^ This problem could be solved by combining our present protocol with immunoaffinity extraction.^23^

**Table 4:** LLOQs for VD metabolites with DAPTAD derivatization in SRM972a level 2 serum

	LLOQ (ng/mL)
25(OH)D_3_	0.12
25(OH)D_2_	0.045
3‐*epi*‐25(OH)D_3_	0.065
24,25(OH)_2_D_3_	0.072

LLOQ, lower limit of quantification.

## 4 CONCLUSIONS

We screened several diene groups to develop the caged Cookson‐type reagent, DAP‐PA, which was stable enough for mid‐ to long‐term storage in solution. Highly stable reagents are essential for data reproducibility in clinical laboratories. The stability of the reagents in a solution is guaranteed by the production of pure products, which is generally achieved by crystallization, and the caged DAPTAD is easy to crystallize, which is a strong advantage in terms of quality control. In addition, from a practical viewpoint, the caged DAPTAD is available in large quantities, and thus market supply is stable and ample. This advantage will contribute to the field of VD detection and quantification in clinical laboratories, and thus to the broader use of clinical mass spectrometry.^24^

## Supporting information

Figure S1. ^1^H‐NMR spectra of the aromatic region of DAPTAD in ethyl acetate‐*d*_8_. DAPTAD signals assigned to aromatic protons are indicated by arrows.
Figure S2. ^1^H‐NMR spectrum of DAP‐PA in CDCl_3_.
Figure S3. ^13^C‐NMR spectrum of DAP‐PA in CDCl_3_.
Figure S4. IR spectrum of DAP‐PA.
Figure S5. ^1^H‐NMR spectrum of DAP‐DP in CDCl_3_.
Figure S6. ^13^C‐NMR spectrum of DAP‐DP in CDCl_3_.
Figure S7. IR spectrum of DAP‐DP.
Figure S8. HPLC chromatograms of the retro‐DA reaction products.
Figure S9. Peak area of DAPTAD‐derivatized VD metabolites on SRM chromatograms. The reaction condition was 0.25 mg/mL in ethyl acetate for 60 min (n=2). Data were obtained using a Waters Xevo TQ‐XS mass spectrometer.
Figure S10. Peak area of DAPTAD‐derivatized VD metabolites on SRM chromatograms. The reaction condition was 0.25 mg/mL in ethyl acetate at 80°C (n=2). Data were obtained using a Waters Xevo TQ‐XS mass spectrometer.
Figure S11. Peak area of DAPTAD‐derivatized VDs on SRM chromatograms. The reaction was performed at 80°C for 15 min (n=2). Data were obtained by using a Waters Xevo TQ‐XS mass spectrometer.
Table S1. Kinetics of anthracene analogues in ethyl acetate at 70°C.
Table S2. Solvent effect for the retro‐DA reaction of DAP‐PA at 70°C.
