# Supplementary material for: A novel caged Cookson‐type reagent toward a practical vitamin D derivatization method for mass spectrometric analyses
Source: Rapid Commun Mass Spectrom. 2020 Feb 19;34(7):e8648. doi: 10.1002/rcm.8648 (PMC7064983; doi:10.1002/rcm.8648)
Supplement: Supplementary file 1 — Figure S1. 1H‐NMR spectra of the aromatic region of DAPTAD in ethyl acetate‐d 8. DAPTAD signals assigned to aromatic protons are indicated by arrows. Figure S2. 1H‐NMR spectrum of DAP‐PA in CDCl3. Figure S3. 13C‐NMR spectrum of DAP‐PA in CDCl3. Figure S4. IR spectrum of DAP‐PA. Figure S5. 1H‐NMR spectrum of DAP‐DP in CDCl3. Figure S6. 13C‐NMR spectrum of DAP‐DP in CDCl3. Figure S7. IR spectrum of DAP‐DP. Figure S8. HPLC chromatograms of the retro‐DA reaction products. Figure S9. Peak area of DAPTAD‐derivatized VD metabolites on SRM chromatograms. The reaction condition was 0.25 mg/mL in ethyl acetate for 60 min (n=2). Data were obtained using a Waters Xevo TQ‐XS mass spectrometer. Figure S10. Peak area of DAPTAD‐derivatized VD metabolites on SRM chromatograms. The reaction condition was 0.25 mg/mL in ethyl acetate at 80°C (n=2). Data were obtained using a Waters Xevo TQ‐XS mass spectrometer. Figure S11. Peak area of DAPTAD‐derivatized VDs on SRM chromatograms. The reaction was performed at 80°C for 15 min (n=2). Data were obtained by using a Waters Xevo TQ‐XS mass spectrometer. Table S1. Kinetics of anthracene analogues in ethyl acetate at 70°C. Table S2. Solvent effect for the retro‐DA reaction of DAP‐PA at 70°C. [file RCM-34-e8648-s001.docx]

**Supplementary Material**

A novel caged Cookson-type reagent toward a practical vitamin D derivatization method for mass spectrometric analyses

Masahiko Seki^a^, Makoto Sato^b^, Masaki Takiwaki^c,d^, Koji Takahashi^c,e^, Yoshikuni Kikutani^c,e^, Mamoru Satoh^d^, Fumio Nomura^d^, Yutaka, Kuroda^e^, and Seketsu Fukuzawa^c,e^*

^a^Medical Association Group, Tokuyama Corporation, 40 Wadai, Tsukuba, Ibaraki 300-4247, Japan

^b^Tsukuba Research Lab, Tokuyama Corporation, 40 Wadai, Tsukuba, Ibaraki 300-4247, Japan

^c^Open Innovation Promotion Department, Management Strategy Planning Division, JEOL Ltd., 3-1-2 Musashino, Akishima, Tokyo 196-8558, Japan

^d^Division of Clinical Mass Spectrometry, Chiba University Hospital, 1-8-1 Inohana, Chuo-ku, Chiba 260-8670, Japan

^e^Department of Biotechnology and Life Science, Faculty of Technology, Tokyo University of Agriculture and Technology, 2-24-16 Naka-cho, Koganei, Tokyo 184-8588 Japan

Tel +81-42-388-7753

Fax: +81-42-546-9732

e-mail: [sfukuzaw@jeol.co.jp](mailto:sfukuzaw@jeol.co.jp), s-fukuzawa@m2.tuat.ac.jp


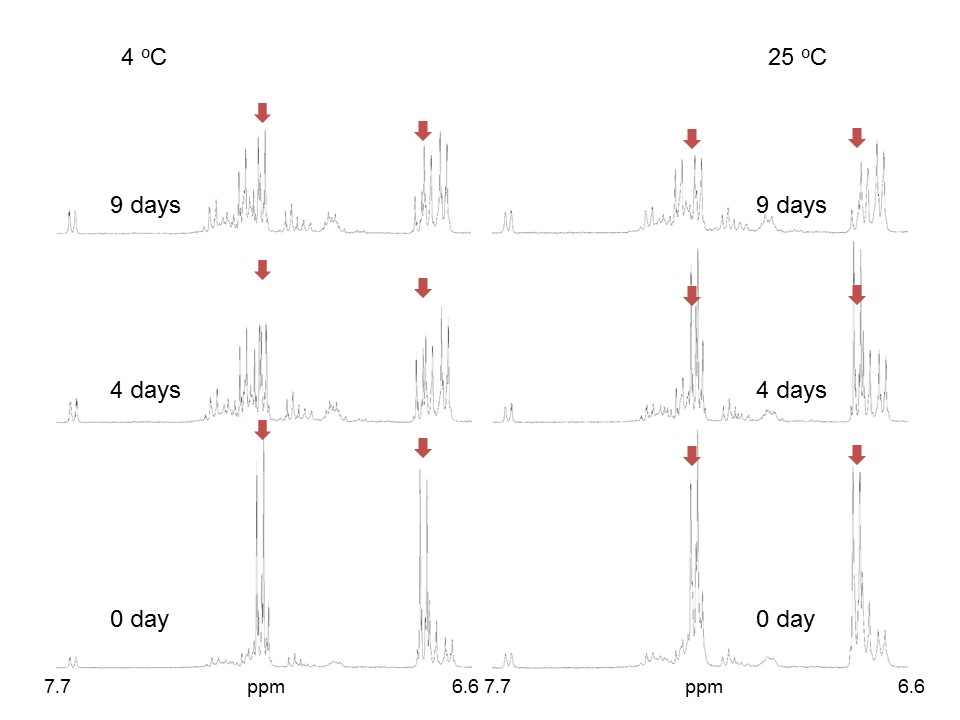


Figure S1. ^1^H-NMR spectra of the aromatic region of DAPTAD in ethyl acetate-*d*_8_. DAPTAD signals assigned to aromatic protons are indicated by arrows.


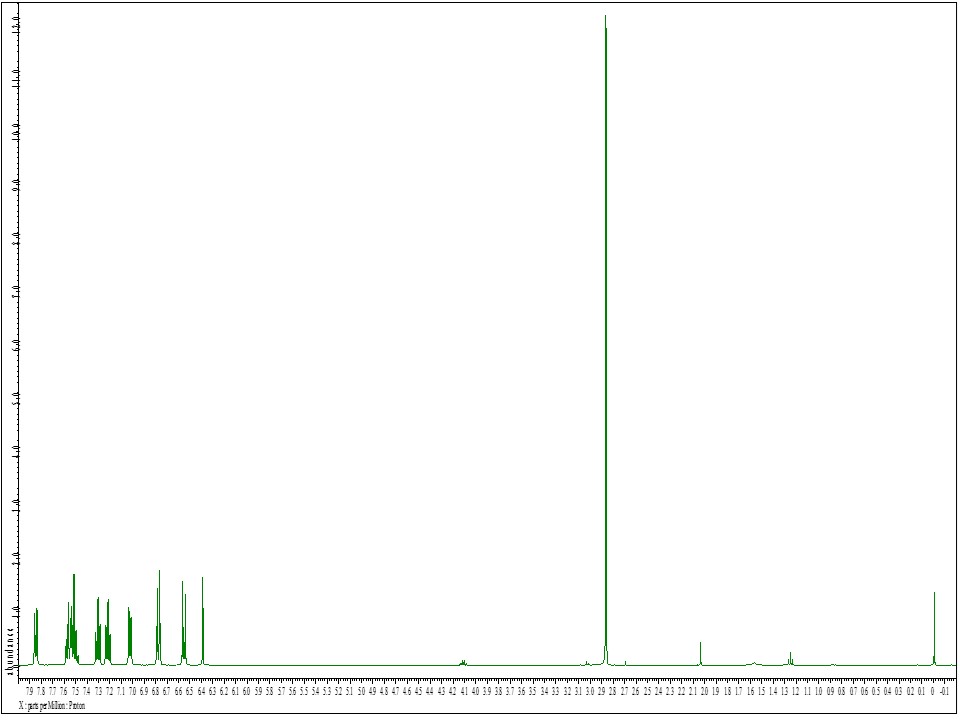
Figure S2. ^1^H-NMR spectrum of DAP-PA in CDCl_3_.


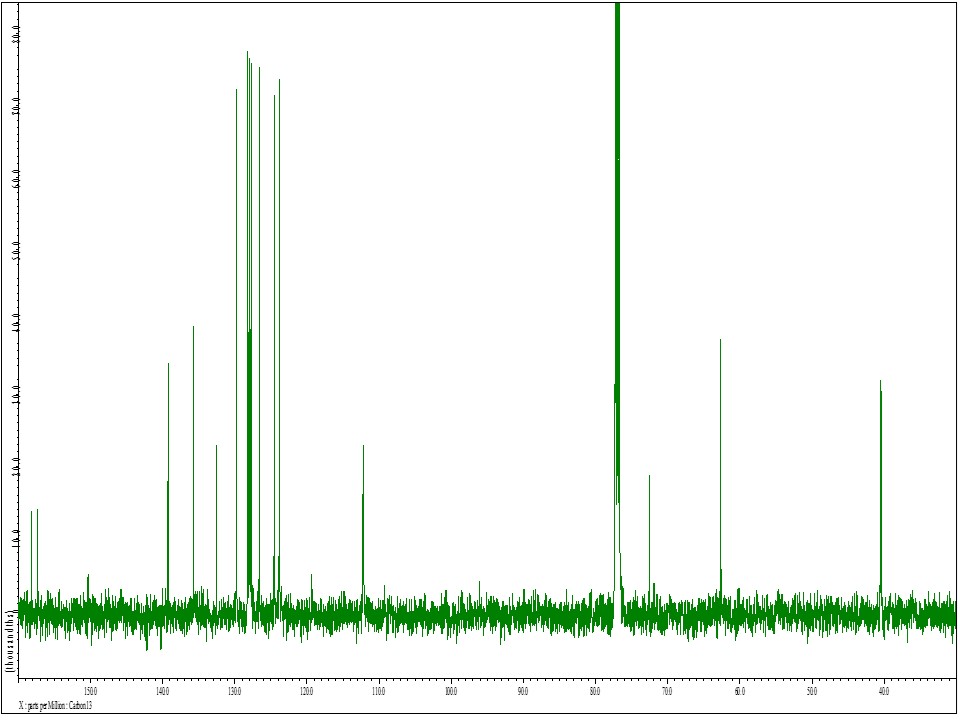


Figure S3. ^13^C-NMR spectrum of DAP-PA in CDCl_3_.


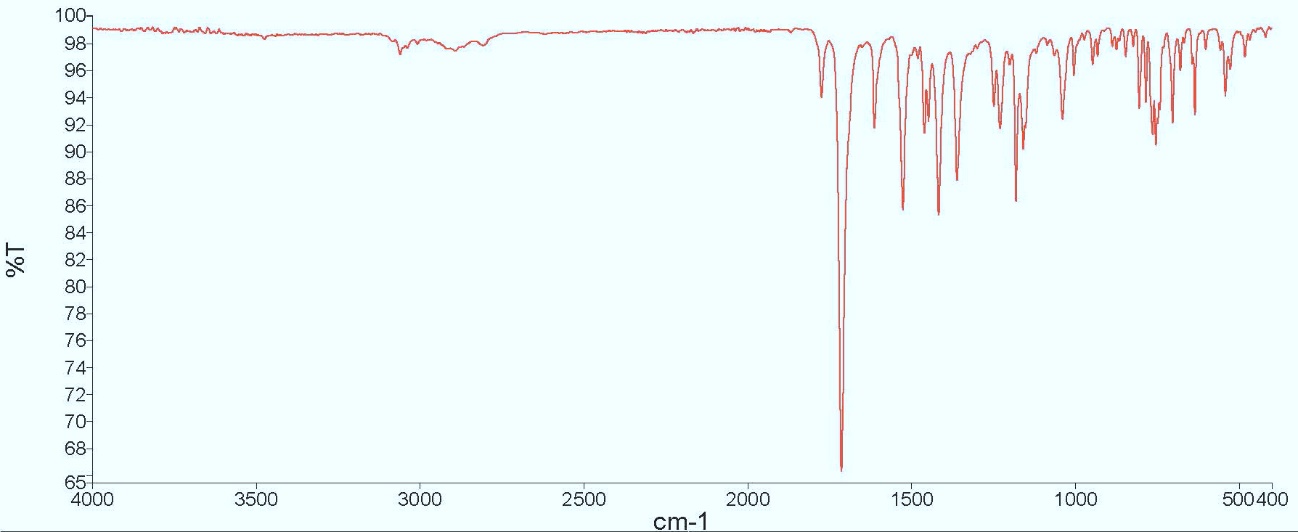


Figure S4. IR spectrum of DAP-PA.


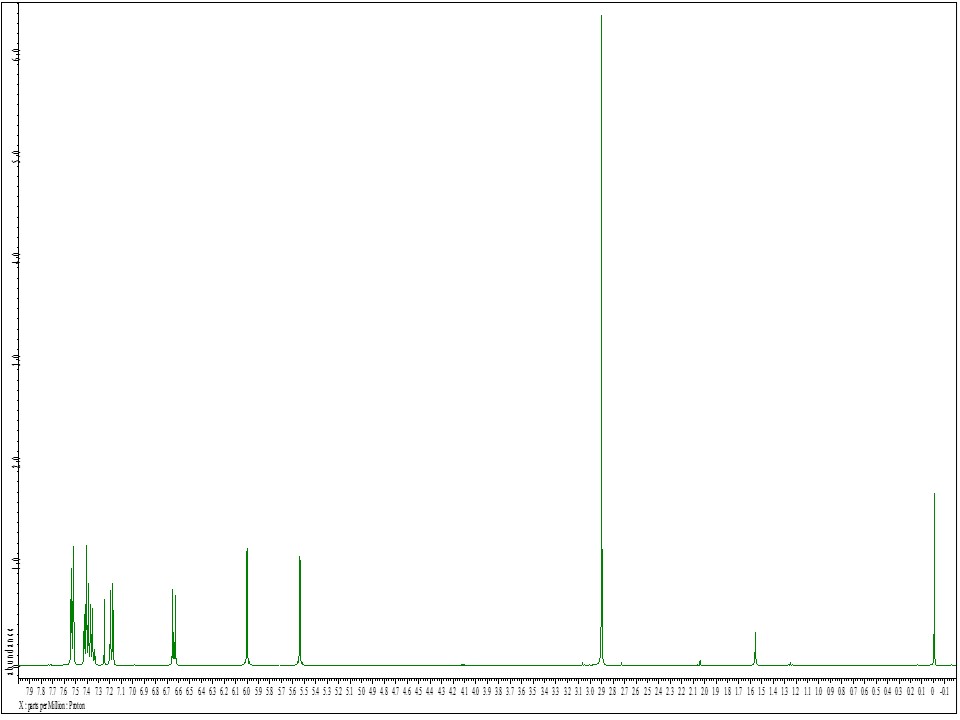
Figure S5. ^1^H-NMR spectrum of DAP-DP in CDCl_3_.


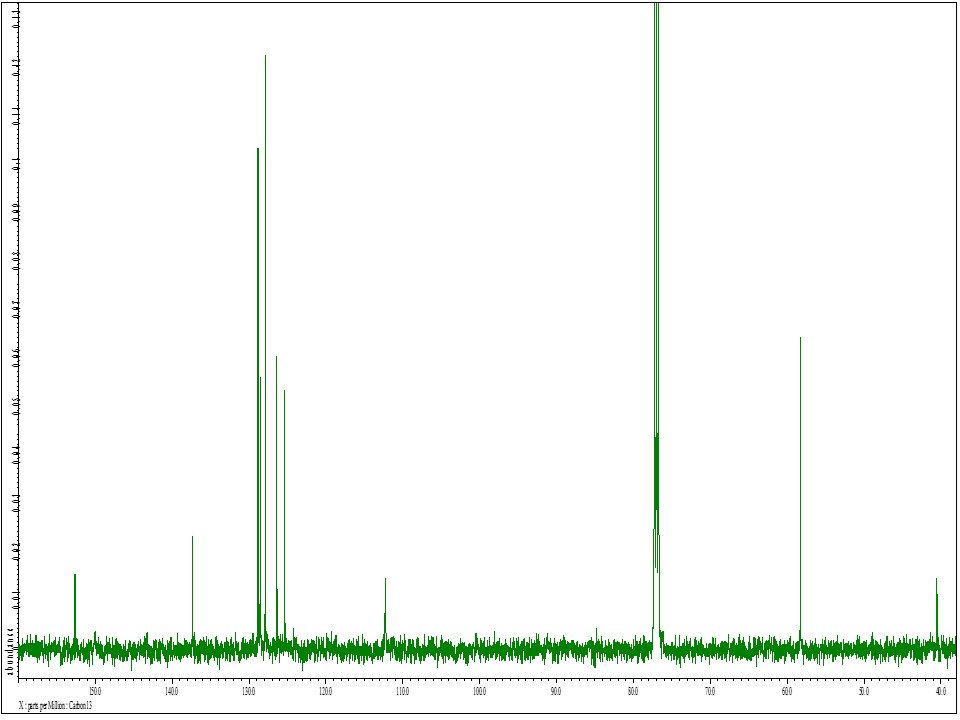


Figure S6. ^13^C-NMR spectrum of DAP-DP in CDCl_3_.


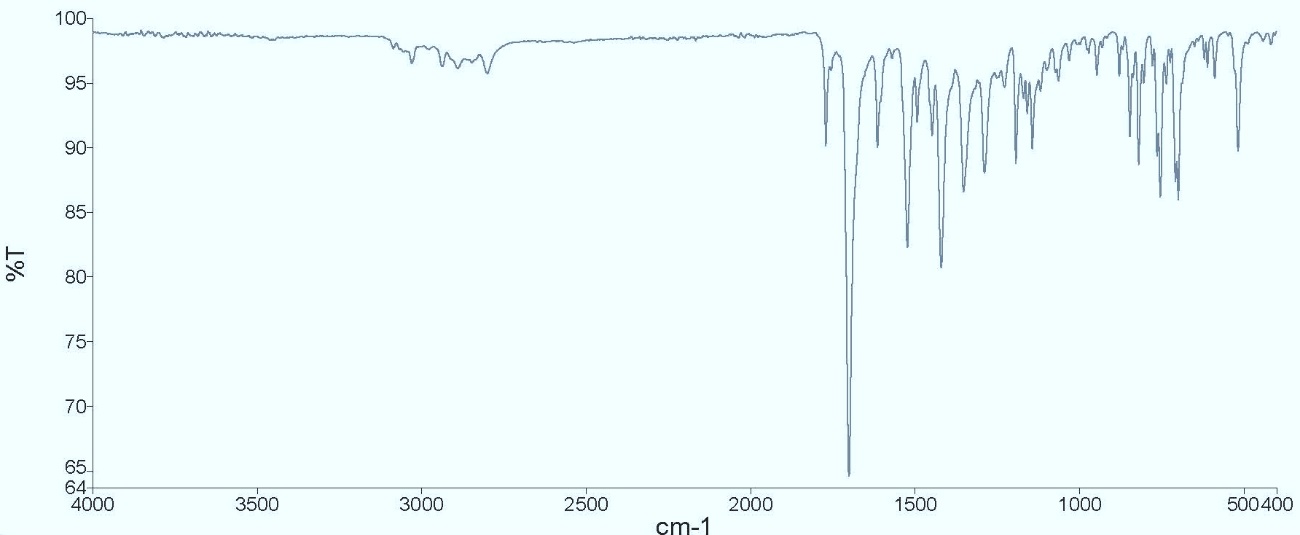


Figure S7. IR spectrum of DAP-DP.

Figure S8. HPLC chromatograms of the retro-DA reaction products.


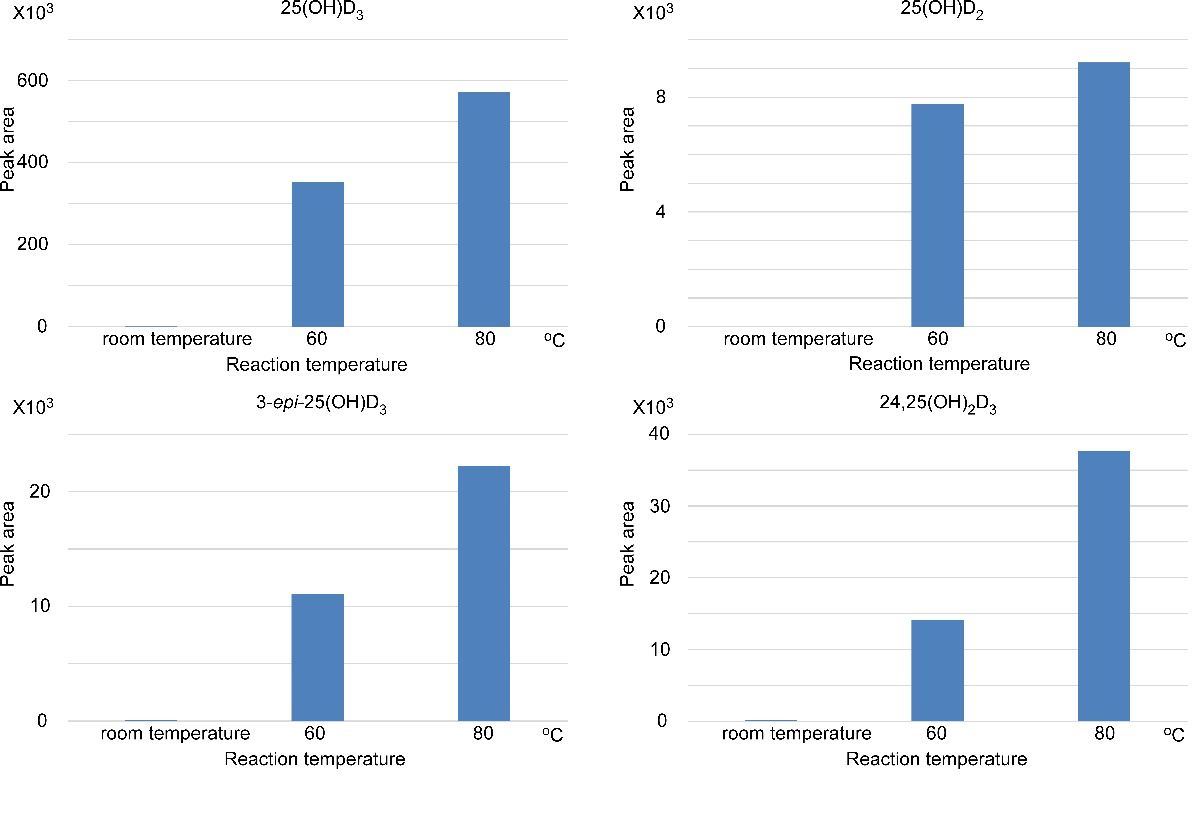


Figure S9. Peak area of DAPTAD-derivatized VD metabolites on SRM chromatograms. The reaction condition was 0.25 mg/mL in ethyl acetate for 60 min (n=2). Data were obtained using a Waters Xevo TQ-XS mass spectrometer.


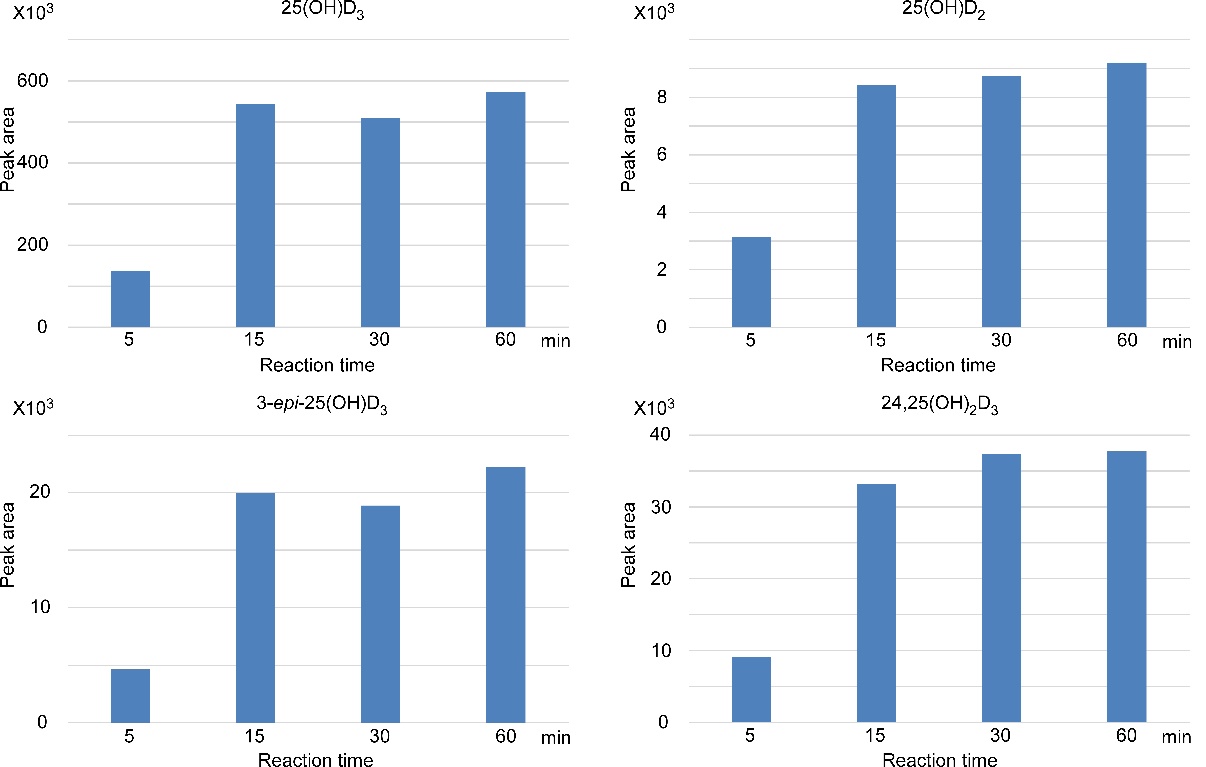


Figure S10. Peak area of DAPTAD-derivatized VD metabolites on SRM chromatograms. The reaction condition was 0.25 mg/mL in ethyl acetate at 80°C (n=2). Data were obtained using a Waters Xevo TQ-XS mass spectrometer.


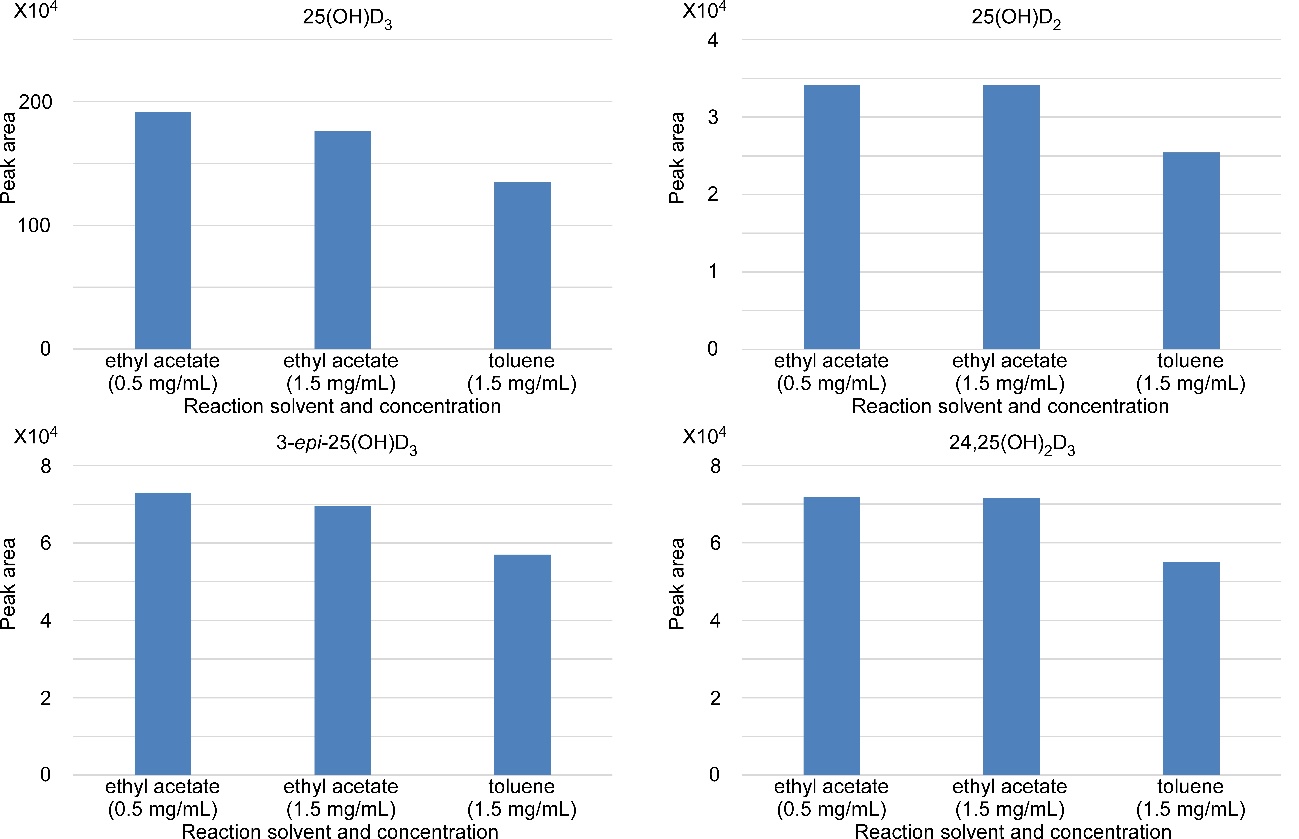


Figure S11. Peak area of DAPTAD-derivatized VDs on SRM chromatograms. The reaction was performed at 80°C for 15 min (n=2). Data were obtained by using a Waters Xevo TQ-XS mass spectrometer.

Table S1. Kinetics of anthracene analogues in ethyl acetate at 70°C.

| Substituent **R** | Substituent constant σ_p_ /– | Rate constant *k*_1_ /h^-1^ |
| --- | --- | --- |
| Methyl | –0.17 | 0^a^ |
| 4-Dimethylaminophenyl | NA | 0^a^ |
| Phenyl (DAP-PA) | –0.01 | 1.01 |
| H | 0.00 | 0^a^ |
| Chloromethyl | +0.12 | 0.02 |
| Br | +0.23 | 0.29 |
| 4-Nitrophenyl | +0.26 | 0.19 |
| Acetyl | +0.50 | 0^a^ |

^a^ No reaction.

Table S2. Solvent effect for the retro-DA reaction of DAP-PA at 70°C.

| Solvent | Boiling point (°C) | Dielectric constant ε_r_ /– | Rate constant *k*_1_ /h^-1^ |
| --- | --- | --- | --- |
| Benzene | 80.1 | 2.28 | 0.82 |
| Toluene | 110.6 | 2.38 | 1.44^a^ |
| Ethylbenzene | 136 | 2.50 | 0.92 |
| *o*-Xylene | 144 | 2.57 | 0.99 |
| Anisole | 154 | 4.33 | 1.73 |
| Cyclopentyl methyl ether | 106 | 4.76 | 0.76 |
| Bromobenzene | 156 | 5.40 | 1.50 |
| Chlorobenzene | 131 | 5.62 | 2.41 |
| Ethyl acatate | 77.1 | 6.02 | 1.01 |
| *o*-Dichlorobenzene | 180.5 | 6.83 | 0.64 |
| Acetonitrile | 82 | 37.5 | 0.89 |

^a^ Unknown signal was observed on HPLC chromatogram.
